# Supplementary material for: Fas2EB112: a tale of two chromosomes
Source: G3 (Bethesda). 2024 Mar 6;14(5):jkae047. doi: 10.1093/g3journal/jkae047 (PMC11075550; doi:10.1093/g3journal/jkae047)
Supplement: jkae047_Supplementary_Data [file jkae047_supplementary_data.zip › Supplemental_Material_Legends_G3-2024-404945.docx]

**Supplemental Material Legends**

**Supplemental Figure 1: A)** A severe popping out phenotype is observed in homozygous mutant clones generated using the Mannheim *Fas2^EB112^* chromosome. Scale bars = 20 μm. **B)** A schematic showing how the two lethal chromosomes were generated through recombination. The original Bloomington *Fas2^EB112^* chromosome is represented in red and the FRT19A chromosome with which it was recombined is in blue. Purple represents sequence that may have come from either of these. At this point in the study we could not distinguish whether the *e(Fas2)^mut^* chromosome was the product of a single crossover event to the left of the *Fas2* locus or two crossovers to the right (see Supplemental Figure 4).

**Supplemental Figure 2: A and B)** Mitotic spindle angles are parallel to the tissue plane in *e(Fas2)^mut^* tissue. Representative image in (A) and quantification in (B). **C)** Fas3 immunoreactivity at follicle cell-cell borders is retained in *e(Fas2)^mut^* tissue. Scale bars in (A) and (C) = 5 μm.

**Supplemental Figure 3: A)** Diagram illustrating splicing variants that generate different Nrg protein isoforms. The position of the YFP insertion (*Nrg^CPTI001714^*) is also shown. **B)** Immunostaining confirms the specificity of the anti-Nrg antibody. Scale bar = 20 μm. **C)** Occasional nondisjunction of the sex chromosomes results in XXY and XO flies, which can be easily distinguished (and therefore excluded) by eye shape and eye color. **D)** Quantification of Nrg::YFP protein expression (as measured by immunoblot band intensity) across samples shows that it is enhanced in samples lacking Nrg^167^ and Nrg^180^ (related to Figure 2F). Intensity was measured in four immunoblots based on two lysate preparations. Error bars represent standard deviation.

**Supplemental Figure 4: A)** Expression of Nrg from the Y chromosome rescues the viability of *e(Fas2)^mut^* male flies. Balanced mutant females were crossed to males that have Y chromosomes with a duplication of the X chromosome that includes *Nrg*. This strategy allowed for the appearance of *Nrg^14^* and *e(Fas2)^mut^* male progeny. **B)** Single nucleotide polymorphisms (compared to the dm6 reference genome) reveal sequence similarity between the Mannheim *Fas2^EB112^* chromosome and the Bloomington *Fas2^EB112^* chromosome, and also similarity between the Bloomington *Fas2^EB112^* chromosome and the *e(Fas2)^mut^* chromosome. The latter comparison reveals extensive similarity over a region that includes *Nrg*. Both nucleotide position and a cytological map are shown for reference.

**Supplemental Movie 1:** Genetic disruption of both *Fas2* (knockdown driven by Traffic jam-GAL4) and *e(Fas2)* (*e(Fas2)^mut^* clones marked by the absence of RFP, in magenta) causes a severe popping out phenotype. The video is a z-axis fly-through of a stack of images spaced 0.5 μm apart. Actin (phalloidin) is revealed in orange and DNA (DAPI) in cyan.
