# Supplementary figures and images for: Fas2EB112: a tale of two chromosomes
Source: G3 (Bethesda). 2024 Mar 6;14(5):jkae047. doi: 10.1093/g3journal/jkae047 (PMC11075550; doi:10.1093/g3journal/jkae047)

SUPPLEMENTAL FIGURE 1 - Finegan *et al.*

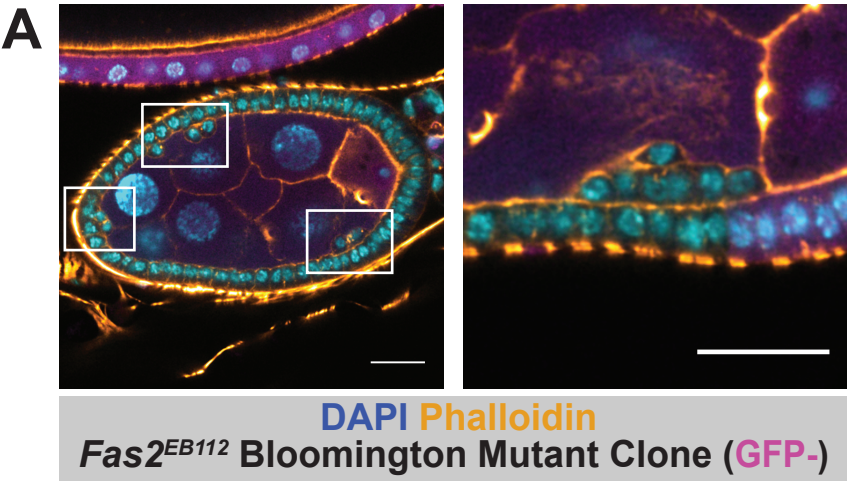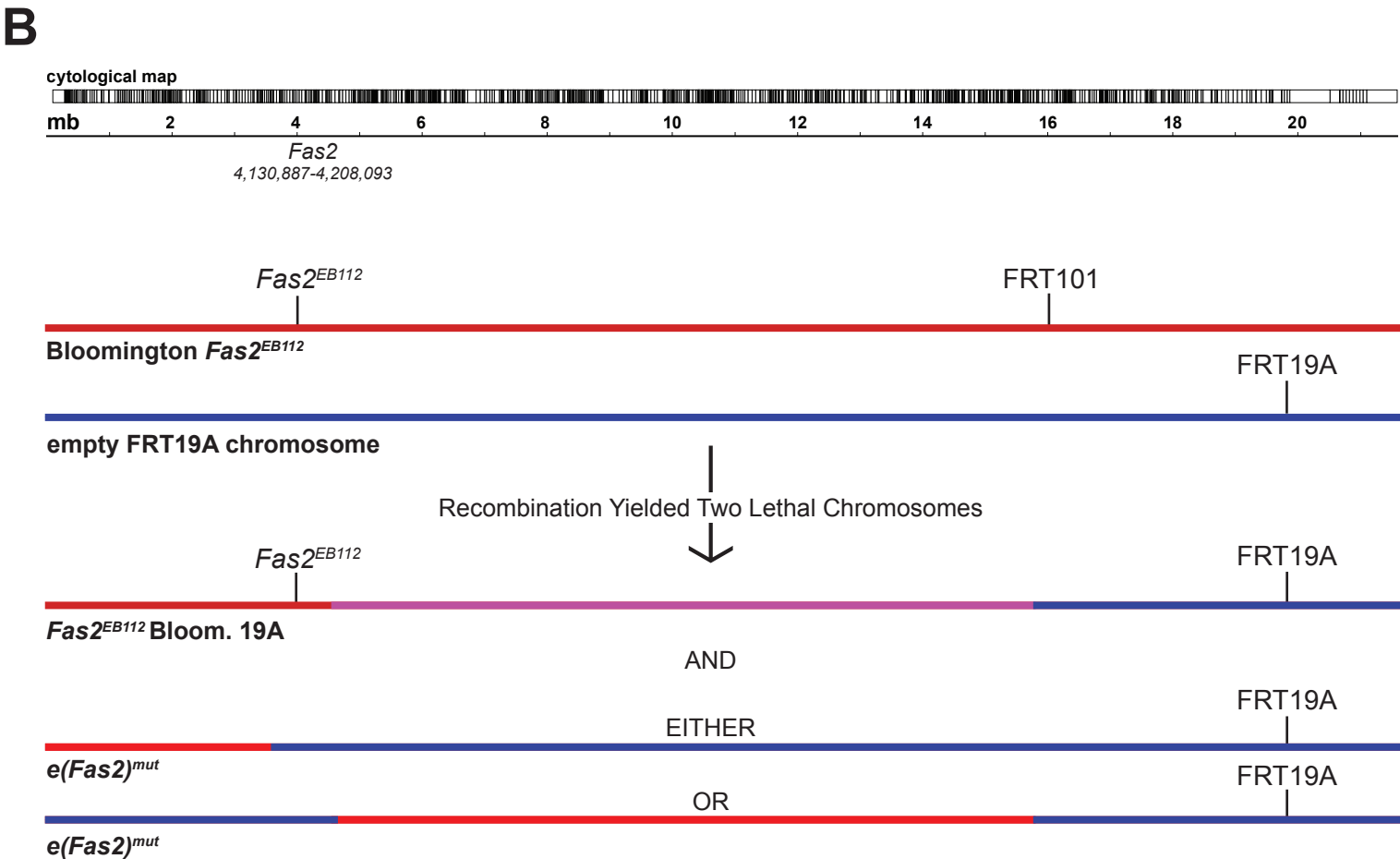

Supplement: jkae047_Supplementary_Data [file jkae047_supplementary_data.zip › Supplemental_Figure_1_G3-2024-404945.pdf]

SUPPLEMENTAL FIGURE 2 - Finegan *et al.*

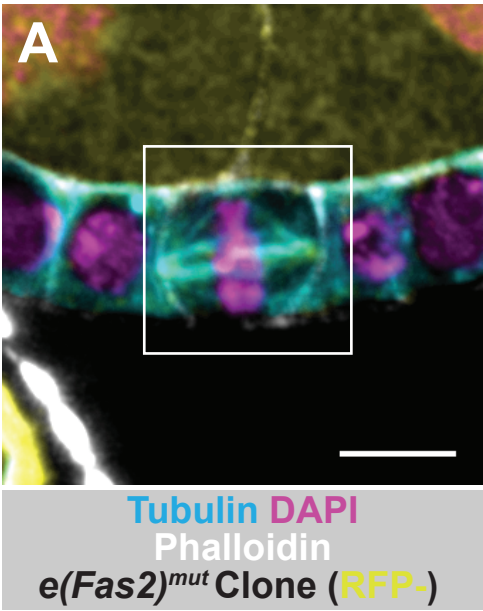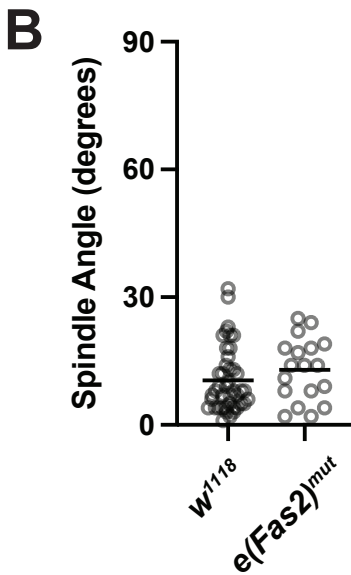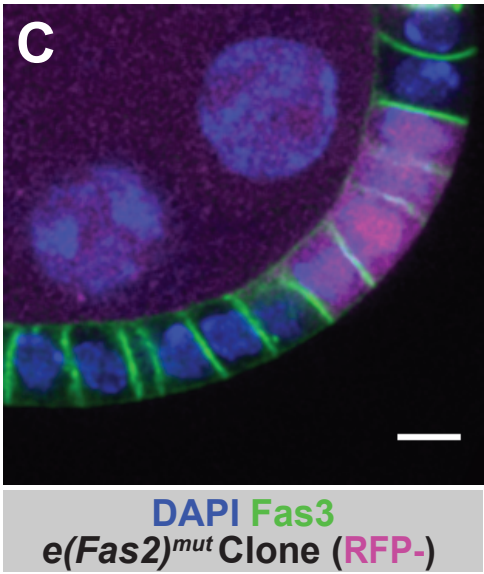

Supplement: jkae047_Supplementary_Data [file jkae047_supplementary_data.zip › Supplemental_Figure_2_G3-2024-404945.pdf]

### SUPPLEMENTAL FIGURE 3 Finegan *et al.*

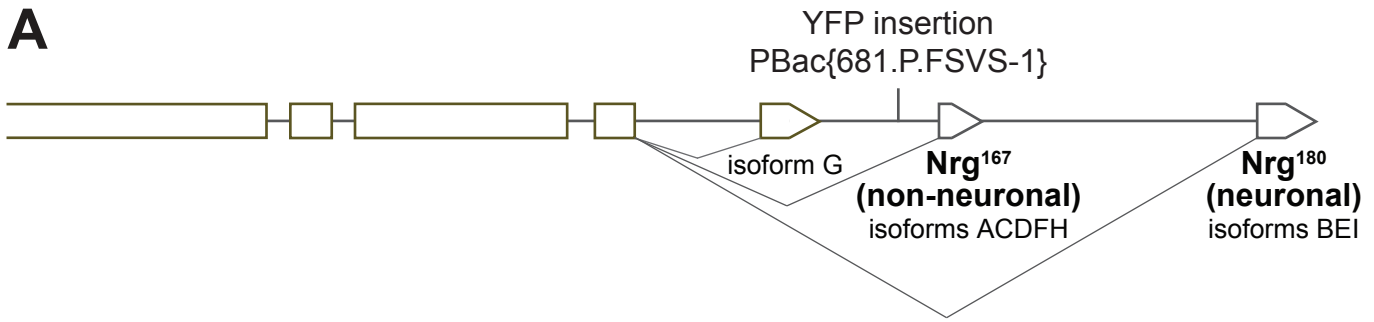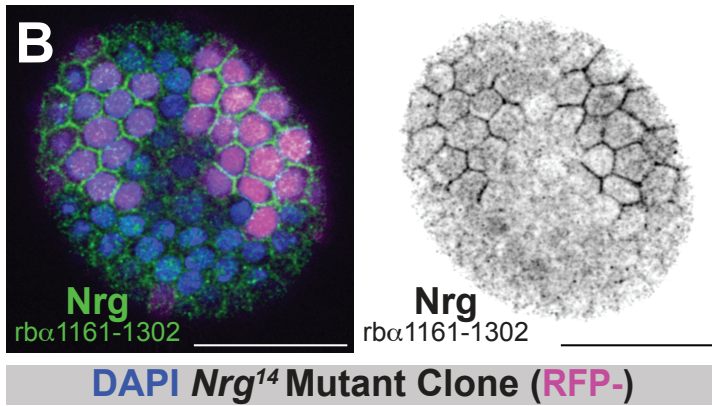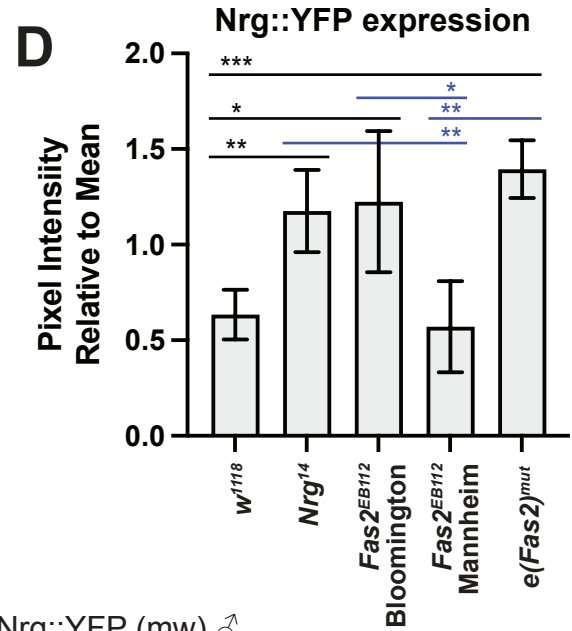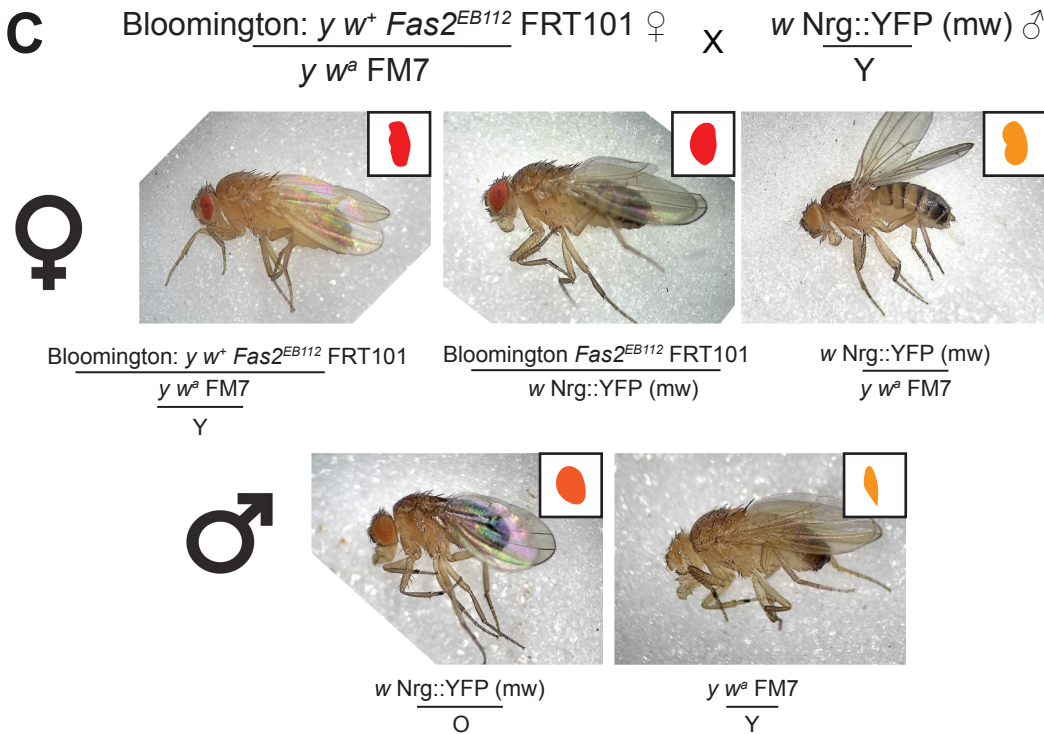

Supplement: jkae047_Supplementary_Data [file jkae047_supplementary_data.zip › Supplemental_Figure_3_G3-2024-404945.pdf]

# SUPPLEMENTAL FIGURE 4 - Finegan *et al.*

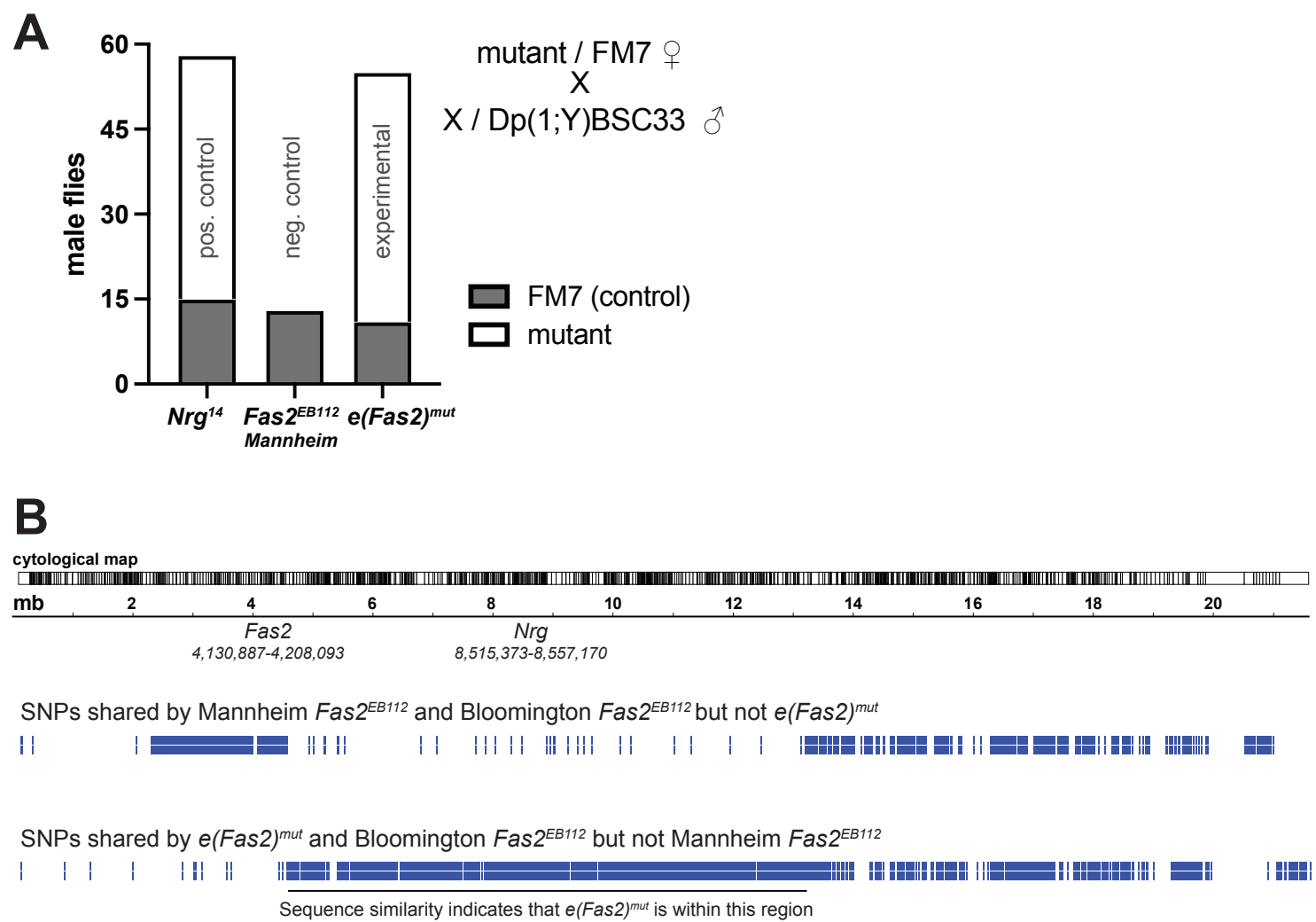

Supplement: jkae047_Supplementary_Data [file jkae047_supplementary_data.zip › Supplemental_Figure_4_G3-2024-404945.pdf]
